# Diversity of Microglia-Derived Molecules with Neurotrophic Properties That Support Neurons in the Central Nervous System and Other Tissues

**DOI:** 10.3390/molecules29235525

**Published:** 2024-11-22

**Authors:** Kennedy R. Wiens, Naved Wasti, Omar Orlando Ulloa, Andis Klegeris

**Affiliations:** Laboratory of Cellular and Molecular Pharmacology, Department of Biology, University of British Columbia, Okanagan Campus, Kelowna, BC V1V 1V7, Canada; kennedywiens@gmail.com (K.R.W.); omar.oulloa@gmail.com (O.O.U.)

**Keywords:** activin A, colony-stimulating factor (CSF)-1, fibroblast growth factor (FGF)-2, growth/differentiation factor (GDF)-15, insulin-like growth factor (IGF)-2, interleukin (IL)-34, leukemia inhibitory factor (LIF), oncostatin M (OSM), neurodegenerative diseases, neurological disorders

## Abstract

Microglia, the brain immune cells, support neurons by producing several established neurotrophic molecules including glial cell line-derived neurotrophic factor (GDNF) and brain-derived neurotrophic factor (BDNF). Modern analytical techniques have identified numerous phenotypic states of microglia, each associated with the secretion of a diverse set of substances, which likely include not only canonical neurotrophic factors but also other less-studied molecules that can interact with neurons and provide trophic support. In this review, we consider the following eight such candidate cytokines: oncostatin M (OSM), leukemia inhibitory factor (LIF), activin A, colony-stimulating factor (CSF)-1, interleukin (IL)-34, growth/differentiation factor (GDF)-15, fibroblast growth factor (FGF)-2, and insulin-like growth factor (IGF)-2. The available literature provides sufficient evidence demonstrating murine cells produce these cytokines and that they exhibit neurotrophic activity in at least one neuronal model. Several distinct types of neurotrophic activity are identified that only partially overlap among the cytokines considered, reflecting either their distinct intrinsic properties or lack of comprehensive studies covering the full spectrum of neurotrophic effects. The scarcity of human-specific studies is another significant knowledge gap revealed by this review. Further studies on these potential microglia-derived neurotrophic factors are warranted since they may be used as targeted treatments for diverse neurological disorders.

## 1. Introduction

### 1.1. Microglial Phenotypes and Secretome

Microglia, the immune cells of the brain, participate in neurodevelopment and support central nervous system (CNS) homeostasis. They maintain functional neurons, the signaling cells of the brain, by secreting neurotrophic factors. Microglia also contribute to the clearance of debris and pathogens by phagocytosis and release of cytotoxins and orchestrating neuroimmune responses by secreting immunomodulatory molecules (reviewed in [1,2,3,4]). Microglia are capable of producing a large number of molecules that act as immunomodulators, cytotoxins, or neurotrophic factors. The spectrum of these molecules released by microglia at any given time—also known as the secretome—depends on the functional status of the cell and its microenvironment (reviewed in [5,6]).

Microglia display phenotypic plasticity by altering their functions and morphology depending on their surrounding environment, including extracellular matrix, stimulants, inhibitors, and other cell types they encounter (reviewed in [7]). Under physiological conditions, surveying microglia typically exhibit ramified morphology and possess both primary and secondary surveillant processes. Pathological conditions such as those that develop in neurodegenerative diseases, neurotrauma, or infection provoke microglia to adopt a reactive phenotype characterized by amoeboid morphology with retracted processes, altered expression of membrane markers, and upregulated secretory activity (reviewed in [8]). Reactive microglia can exhibit overall pro-inflammatory or anti-inflammatory activity involving the release of distinct sets of molecules, which previously has been referred to as the M1 and M2 polarization, respectively; however, recent findings indicate that multiple intermediate and alternative to M1 and M2 functional states of microglia exist, compelling the scientific community to shift towards a multidimensional concept of microglial reactivity (reviewed in [9]). The microglial secretome is highly dependent on their phenotypic state. Under specific conditions, it may include a broad range of pro-inflammatory and neurotoxic molecules that contribute to neuronal death and the propagation of neuroinflammation (reviewed in [6]). Conversely, activation of inflammation-resolving pathways in microglia results in the release of anti-inflammatory and neurotrophic factors (reviewed in [10]). Our review illustrates that similar to their secretion of neurotoxins, reactive microglia are likely to secrete a mixture of neurotrophic molecules, the composition of which depends on the species, nature of the stimulating agent, and the surrounding microenvironment.

### 1.2. Effect of Neurotrophic Factors

The growth, differentiation, and survival of developing and mature neurons are all modulated by tightly controlled exposure to neurotrophic factors (reviewed in [11]). New neurons appear most rapidly during nervous system development, but this process persists at varying degrees throughout the lifespan (reviewed in [12]). Existing cells sprout and extend neurites, which enables them to establish new connections with other neurons, aiding in synaptogenesis and neurotransmission (reviewed in [13]). Various CNS insults can lead to neuronal death, but if favorable conditions are restored, neurons are able to repair damage to their cell body and processes as well as re-establish lost synaptic connections (reviewed in [14]). For the purposes of this review, molecules are considered neurotrophic if they have the capacity to facilitate at least one of the following processes: neuronal proliferation, differentiation of neuronal precursors, growth of pre-existing and new neuronal projections, and survival or recovery after neuronal damage.

### 1.3. Microglia-Derived Neurotrophic Factors

**Figure 1:**
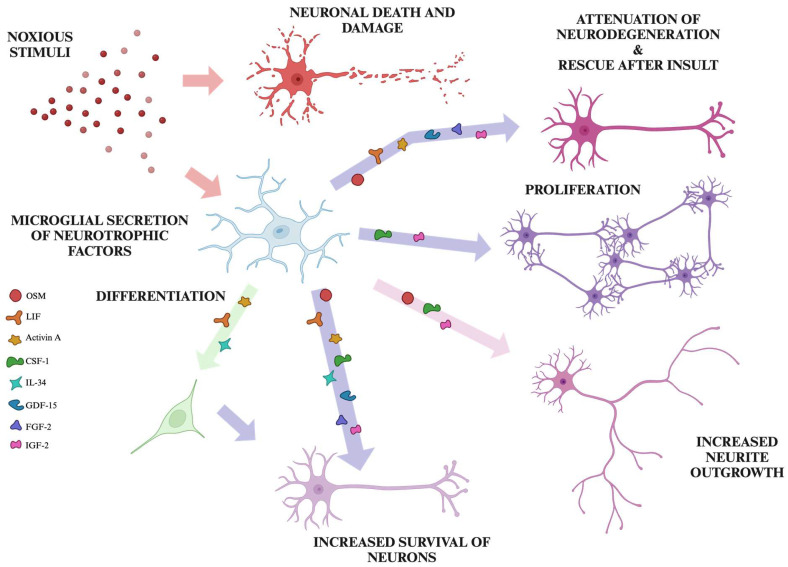
Microglia-derived molecules with potential neurotrophic properties.

Previous literature includes a number of extensive reviews that are typically focused on a subset of microglia-derived proteins with proven and widely accepted neurotrophic functions. They include brain-derived neurotrophic factor (BDNF), nerve growth factor (NGF), glial cell line-derived neurotrophic factor (GDNF), neurotrophin (NT)-3, and NT-4/5 (reviewed in [15,16,17,18]). However, there are other proteins and peptides that are not yet considered prototypical microglia-derived neurotrophic factors, but experimental evidence has been collected indicating they (1) are secreted by either surveying or reactive microglia, (2) interact with neurons by binding to a corresponding receptor or receptors, and (3) this interaction leads to neurotrophic effects under certain experimental conditions. In this review, we consider the following eight cytokines secreted by microglia that bind to distinct neuronal targets and demonstrate neurotrophic effects in vitro or in the CNS: oncostatin M (OSM), leukemia inhibitory factor (LIF), activin A, colony-stimulating factor (CSF)-1, interleukin (IL)-34, growth/differentiation factor (GDF)-15, fibroblast growth factor (FGF)-2, and insulin-like growth factor (IGF)-2 (Figure 1).

## 2. Oncostatin M

### 2.1. Overview of Structure and Function

Oncostatin M (OSM) is a 24 kDa member of the IL-6 cytokine family. Despite being discovered more than 35 years ago (reviewed in [19]), novel functions of OSM and other members of the IL-6 family are still being revealed. Murine OSM (mOSM) and human OSM (hOSM) share only 49% amino acid sequence homology (reviewed in [20]). Furthermore, hOSM does not engage the same receptor as mOSM when administered in murine models, indicating that the biological activity of OSM is species-specific; thus, hOSM and mOSM cannot be used interchangeably in experimental studies across different species (reviewed in [21]). In the periphery, OSM plays established roles in osteogenesis and hematopoiesis (reviewed in [22]). In the CNS, OSM has diverse functions, including remodeling of the extracellular matrix, inhibition of brain tumor formation, and maintaining homeostasis of neural precursor cells in the subventricular nucleus and dentate gyrus [23,24,25]. While these and several other studies suggest that OSM is likely a neuroprotective molecule, its neurotrophic activity in both murine and human models requires more detailed investigations.

### 2.2. Expression and Secretion by Microglia

Monocytes, macrophages, dendritic cells, and neutrophils are the primary producers of OSM in the peripheral tissues of both mice and humans [26]. Hematopoietic cells found in bone marrow also constitutively secrete OSM [27]. Microglia are the primary source of OSM in both the murine and human CNS. Table 1 illustrates that transcriptomic analysis of primary mouse microglia shows an average of 209.9 OSM fragments per kilobase of transcript per million mapped reads (FPKM), which is a relatively high value. Human microglia harvested from brain tissue collected during surgery show a much lower 3.2 FPKM for OSM. While this expression level is low, microglia are likely the only significant source of OSM in the human CNS [28]. Table 1 displays FPKM values for all cytokines covered in this review and their receptors for microglia and neurons, respectively. FPKM is a validated approach used to compare levels of mRNA expression within a single sample; however, it is important to note this technique should not be used when comparing values from different samples or sources (reviewed in [29,30,31]).

Upregulation of both OSM mRNA and protein has been observed in murine microglia under specific conditions. For example, treatment with prostaglandin E_2_ (PGE_2_) and infection with EcoHuman immunodeficiency virus (HIV)—a chimeric HIV-1 designed to emulate the pathology associated with HIV in a murine model—upregulate OSM mRNA and protein in BV-2 murine microglia [32,33]. Similar phenomena are observed in PGE_2_-stimulated THP-1 human monocytic cells, which are often used to model human microglia [33]. Human U-937 monocytic cells, which are also microglia-like, exhibit upregulated OSM mRNA expression following exposure to granulocyte-macrophage colony-stimulating factor (GM-CSF), lipopolysaccharide (LPS), and phorbol-12-myristate-13-acetate (PMA) [34].

In a murine model of spinal cord injury (SCI), OSM mRNA expression is shown to be upregulated compared to spinal cords from uninjured animals [35]. Induction of OSM protein expression in humans is demonstrated in biopsy and autopsy samples of brain tissues from multiple sclerosis (MS) patients. OSM protein has been colocalized with several cell types, including reactive microglia, hypertrophic astrocytes, and infiltrating leukocytes within the MS lesions that undergo active demyelination. In contrast, OSM protein is undetectable in control brains with no MS pathology [35].

### 2.3. Neuronal Targets

The OSM receptor (OSMR) complexes consist of two transmembrane protein subunits [36]. One of them is the glycoprotein (gp)130α, and the other can be either an OSMRß or leukemia inhibitory factor receptor (LIFR)ß subunit [20]. The OSMR complexes are species-specific, but all share the common gp130α subunit. mOSM binds to an OSMR complex consisting of a gp130α subunit and an OSMRß subunit [37]. hOSM possesses the distinctive ability to recruit two different receptor complexes, known as OSMR1 and OSMR2 [20]. OSMR1 consists of a gp130α and a leukemia inhibitory factor receptor (LIFR)ß subunit, while OSMR2 is composed of the same subunits as the murine OSMR [36]. In mice, the gp130/LIFRß complex, which is equivalent to human OSMR1, does not function as a secondary OSMR as it does in humans. One of the primary limitations in the current OSM-focused studies is that the human protein cannot be used in mouse models, as the gp130/hOSM receptor-ligand complex does not engage the OSMRß subunit, but rather the LIFRß subunit. Thus, studies in which hOSM is administered in mice models are not considered in this review. Interestingly, two distinct OSMR complexes are present in rat cells, more closely resembling the human receptors. hOSM binds to the rat OSMR1, comprised a gp130α subunit and LIFRß subunit, which elicits responses similar to those seen in human cells (reviewed in [20]). Unless otherwise specified, OSM and other cytokines discussed in this review are used in a species-specific manner. In the text below, we identify the species of origin for the cytokines only in cases where they originate from a species different from the experimental model in which they are utilized.

OSMRß has low tissue specificity in the periphery and its mRNA is expressed in many organs [38,39]. Primary neurons from both mice and humans are shown to express all OSMR subunits (see Table 1). Low-level OSMRß mRNA expression is demonstrated in most regions of mouse CNS, including the forebrain, cortex, midbrain, hindbrain, and spinal cord [40]. Contrarily, the expression of OSMRß in human brains at either the mRNA or protein level has not been documented, and further research is required to address this knowledge gap.

gp130α mRNA and protein are present ubiquitously in the peripheral organs of mice and humans (reviewed in [41]). gp130α mRNA is also expressed by most CNS cell types in both mice and humans [28,42]. The ubiquitous presence of gp130α mRNA has been reported for both murine and human excitatory and inhibitory neurons [43,44], and in C57BL/6 mice, there is evidence of gp130 protein expression by CNS neurons [45]. LIFR mRNA is expressed in the olfactory bulb, cerebral cortex, hippocampus, thalamus, and lateral habenular nucleus of adult rats [46]. Based on the observed staining pattern, the authors suggest that the primary cellular source of LIFR in rats is neurons. In humans, neuronal expression of LIFR in the brains of healthy controls is highest in the hippocampus and in the anterior cingulate cortex. In the brains of Alzheimer’s disease (AD) and Parkinson’s disease (PD) patients, LIFR expression is higher than in the control brains. Immunohistochemistry demonstrates co-localization of this protein with mainly hippocampal neurons in AD and anterior cingulate cortex neurons in PD [47].

### 2.4. Neurotrophic Effects

In cultured primary murine cortical neurons, death caused by *N*-methyl-d-aspartate (NMDA)-induced excitotoxic injury is reduced by 50% when cells are treated with OSM. In vitro, the presence of OSM also leads to a moderate 20–30% reduction in cortical neuron death in response to kainic acid (KA) and α-amino-3-hydroxy-5-methyl-4-isoxazole propionic acid (AMPA)-induced injuries [48]. B-27 supplement-deprived primary murine cortical neurons exposed to OSM demonstrate increased viability compared to untreated cells, indicating that OSM may possess neuroprotective activity. Enhanced axonal growth is also observed, supporting the role of OSM in promoting neurite regeneration [49].

Mice with excitotoxic brain injury induced by intrastriatal injection of NMDA demonstrate decreased lesion size in the neuron-dense striatum when co-injected with OSM in comparison to mice not receiving OSM [48,50]. In a murine SCI model, OSM facilitates the recovery of neurons, prevents neuronal death, and promotes neurite outgrowth. Additionally, a decrease in lesion size at the site of the SCI is observed, accompanied by upregulated mRNA expression of both OSM and OSMRß [49]. In a model of MS where mice are fed a 0.2% cuprizone diet to induce demyelination, knocking out the OSMR gene results in exacerbated demyelination of neuronal fibers in the corpus callosum compared to wild-type (WT) mice. This highlights the role of OSM and OSMR signaling in slowing demyelination. Furthermore, the stereotactic injection of OSM-encoding lentiviral factors into the corpus callosum strongly limits demyelination in this murine model of MS [51]. In a murine model of ischemic stroke induced by the left middle cerebral artery occlusion (MCAO), neurological deficits of greater severity including tremor, impaired reflexes, and paralysis are observed in OSMRß-knockout mice compared to WT animals after both 24 and 72 h. Similarly, in a rat model of ischemic stroke induced by left MCAO, intracerebral injection of hOSM promotes neuronal survival and reduces the severity of neurological deficits such as impaired reflexes, lost balance, and decreased grip strength compared to animals not treated with this cytokine [52].

Due to the similarity of OSM signaling in rats and humans and the 52% sequence homology between rOSM and hOSM, administration of hOSM in rats could lead to outcomes that are relevant to human pathophysiology (reviewed in [20]). The sole study on OSM protein expression in the human brain concludes that OSM may possess immunoregulatory activity [35]. Even though the authors propose OSM as a potential therapeutic agent for treating neuroinflammatory diseases of the CNS, studies using human-specific cell and tissue models are clearly indicated. Furthermore, the intracellular signaling mechanisms responsible for the neurotrophic effects of OSM need to be established. Future studies should focus on the Janus kinase (JAK)/signal transducer and activator of transcription (STAT) pathway since it is activated by binding of OSM to OSMR complexes in murine neurons [52]. This observation has not yet been confirmed for human neurons, but OSM has been shown to activate both STAT1 and STAT3 DNA binding in primary human astrocytes [53], which may indicate the existence of a similar mechanism in human neurons.

## 3. Leukemia Inhibitory Factor

### 3.1. Overview of Structure and Function

Leukemia inhibitory factor (LIF) is a 20 kDa pleiotropic cytokine belonging to the IL-6 cytokine family. It is originally synthesized as a 202-amino-acid precursor, which is post-translationally cleaved at the N-terminal end to produce the final protein [54]. LIF possesses inconsistent and sometimes contradictory activities depending on the cell type and environment it acts in, making it a molecule of great interest in studies of neurodegenerative disease. Murine LIF (mLIF) and human LIF (hLIF) display 78% amino acid homology and have species-specific receptors that are only 70% homologous [55,56]. When administered to a murine model, hLIF binds to murine LIF receptor (LIFR) complexes and elicits a similar response to that observed in humans. However, mLIF cannot bind to human LIFR complexes; therefore, it does not elicit any response in humans [57]. Both mLIF and hLIF competitively inhibit each other in a reciprocal manner when binding to murine LIFR [58]. Notably, despite only 22% amino acid homology with OSM, LIF has a nearly identical protein structure to that of OSM [55]. In a murine model, the administration of hOSM results in activation of the LIFR complex rather than the OSMR complexes (reviewed in [20]).

As its name suggests, LIF inhibits the proliferation of myeloid leukemia cells and induces their differentiation [56]. Additionally, it plays well-established roles in reproduction and hematopoiesis [59]. In the CNS, LIF modulates neurotransmitter production, causing a shift from catecholamines to acetylcholine. In combination with other factors, it also promotes the differentiation of cultured precursor cells into mature astrocytes or oligodendrocytes (reviewed in [60]).

### 3.2. Expression and Secretion by Microglia

In the periphery, LIF mRNA is expressed by the smooth muscle cells of the urinary bladder and glandular cells of the endometrium in both mice and humans [39,61]. In the murine brain, LIF mRNA is expressed primarily by microglia and astrocytes, whereas in the human brain, it is expressed at very low or undetectable levels under physiological conditions (Table 1) [28,42]. Notably, Müller glia cells of the retina exhibit drastically increased LIF mRNA production in murine models of photoreceptor death [62]. Similarly, treatment with extracellular ATP, an established damage-associated molecular pattern (DAMP), has been shown to upregulate the expression of LIF mRNA by cultured primary murine microglia [63]. LIF mRNA or protein has not been investigated in immortalized human microglial cell lines. In primary human microglia, LIF mRNA is present at very low levels under physiological conditions but can be upregulated in pathologies involving infection, inflammation, or injury. For example, in cultured primary human cortical microglia infected with *Borrelia burgdorferi*, the primary cause of Lyme disease, LIF mRNA expression is drastically increased compared to uninfected cells [64].

### 3.3. Neuronal Targets

The LIFR complex is analogous to OSMR1, but unlike OSM signaling, only one receptor complex interacts with LIF (reviewed in [20]). This complex has the same composition in both mice and humans, existing as a transmembrane receptor consisting of gp130α and LIFRß subunits [57]. Expression of gp130α and LIFRß (Table 1) in both mice and humans is discussed in Section 2.3. LIF binds to neuronal heterodimeric LIFR complexes with high affinity. Following receptor activation, the gp130α subunit phosphorylates JAK which then subsequently phosphorylates STAT3, rendering it able to translocate to the nucleus and modulate transcription [65]. Some of the target genes of JAK-STAT signaling include p21, MYC, nitric oxide (NO) synthase (NOS)2, and suppressor of cytokine signaling (SOCS) (reviewed in [66]).

### 3.4. Neurotrophic Effects

Treatment with exogenous LIF promotes the survival of cultured primary murine neurons isolated from the spinal cord of embryonic day 15 (E15) mice in comparison to both untreated control cultures and cells exposed to LIF in the presence of an anti-LIFR antibody [67]. In rats that have undergone intracerebroventricular (ICV) infusion of LIF for 14 days, expression of choline acetyltransferase (ChAT), the enzyme responsible for acetylcholine synthesis, is retained by 90% in axotomized cholinergic septohippocampal neurons isolated from the medial septal nucleus. In comparison, neurons from control animals infused with a saline vehicle solution maintain only 30% of ChAT expression [68]. In a murine model of pediatric mild traumatic brain injury (TBI), acute intranasal LIF administration twice daily for 3 days is shown to ameliorate neuronal injury, resulting in increased axonal growth and sensorimotor function [69].

Human stem cell lines isolated from CNS tissues and cultured with LIF demonstrate a two-fold increase in neuronal differentiation compared to control cells [70]. The same study also finds that LIF promotes survival and neurite outgrowth of neurons terminally differentiated from both diencephalic and cortical human stem cells. The catecholaminergic SH-SY5Y human neuroblastoma cells treated with LIF show increased rates of survival compared to control cells when subjected to the harmful action of peroxides, tumor necrosis factor (TNF), or hypoxic conditions. In this model, hypoxic insult involves the removal of oxygen for six consecutive hours, followed by a return to physiological conditions. Pretreatment with LIF increases the survival of cells by 53% following hypoxic insult, 65% after treatment with peroxides, and 73% following treatment with TNF compared to control cells [71]. Further research into the responses of human cells and tissues to LIF is required to fully elucidate the neurotrophic properties of this molecule.

## 4. Activin A

### 4.1. Overview of Structure and Function

Activin A is a 26 kDa homodimeric polypeptide belonging to the transforming growth factor (TGF)-ß superfamily (reviewed in [72]). All activins consist of two inhibin ß subunits, which dimerize post-translationally to form the secreted protein. There are four different ß subunits that have been identified in mammals, termed ß_A_, ß_B_, ß_C_, and ß_E_. Activin A consists of two 13 kDa inhibin ß_A_ (INHBA) subunits [73]. Therefore, when discussing mRNA expression, INHBA is used, and for protein, activin A is used. The sequence of INHBA is highly conserved across species. In particular, rats and humans share high amino acid homology [74,75]. As such, cross-species administration is a viable option for studying the biological activity of activin A.

Activin A was discovered in the 1980s and first determined to play a role in reproductive physiology. Later, its contributions to cardiac myogenesis, as well as immune cell migration and tumor immunity were recognized [76,77]. The biological effects of activin A on the CNS neurons were first reported in the 1990s [78]. More recently, its anti-inflammatory effects on microglia-mediated neuroimmune signaling have been elucidated (reviewed in [79]).

### 4.2. Expression of Inhibin ß_A_ and Secretion of Activin A by Microglia

INHBA mRNA is expressed by murine Leydig cells, Sertoli cells, and granulosa cells in the gonads, as well as by gonadotropes, mammotropes, and somatotropes in the pituitary gland [80,81,82,83,84]. Human gonads and pituitary tissues also express INHBA mRNA [39]. Microglia are likely the only significant source of INHBA in the mature human CNS but cultured primary murine microglia express very low or undetectable levels under physiological conditions (Table 1). The toll-like receptor (TLR)4 agonist LPS upregulates INHBA mRNA expression by cultured MG6 murine microglial cells [85]. More generally, activin A protein secretion by cultured primary murine microglia is upregulated in response to treatment with agonists of TLR2, 4, or 9, including LPS [86]. The endogenous mediators or signaling pathways modulating INHBA mRNA or activin A protein levels in human microglia or microglia models have not yet been characterized. While INHBA mRNA and activin A protein are expressed at relatively low or undetectable levels under physiological conditions in murine and human microglia, activin A is upregulated during CNS pathologies or insults. In rats, activin A protein in the nucleus accumbens microglia is increased following a cocaine binge [87]. Additionally, a study conducted by Wilms et al. [88] finds abnormally elevated levels of activin A in the cerebrospinal fluid of patients diagnosed with bacterial meningitis. The authors hypothesize that microglia are likely the primary producers of activin A in this case.

### 4.3. Neuronal Targets

Activin A signals through heteromeric complexes consisting of type 1 and type 2 serine/threonine receptor kinases. The type 1 receptors are activin receptor (ACVR) type 1A (ACVR1A) and ACVR1B, while the type 2 receptors are ACVR2A and ACVR2B [89,90,91]. Murine and human activin A receptors display 99% amino acid homology [92]. In the periphery, mRNAs for all four ACVR subtypes are widely expressed. Particularly high levels are present in epithelial cells of the digestive system and endocrine cells of the pituitary gland in both mice and humans [39,61].

In the CNS, both murine and human-cultured primary cortical neurons express higher levels of activin receptor mRNA than non-neuronal cells. In cultured primary murine and human cortical neurons, ACVR1A, ACVR2A, ACVR1B, and ACVR2B are expressed at varying levels with ACVR2A and ACVR1B being the predominant forms in both these species (Table 1). In vivo presence of ACVR1A and ACVR2A in cortical and hippocampal rat neurons is demonstrated by immunolocalization of the receptors using polyclonal IgG antibodies specific for each receptor subunit [93]. ACVR1B and ACVR2B expression has not been confirmed in murine neurons, while none of the ACVRs have been studied in the human CNS. Initially, activin A binds to either ACVR2A or ACVR2B, which recruit and phosphorylate ACVR1A or ACVR1B in a respective manner, resulting in receptor activation. These kinases then bind and phosphorylate cytosolic Smad2 or Smad3, which forms a complex with Smad4 that translocates to the nucleus to modulate transcription of target genes such as c-Jun *N*-terminal kinase 1(JNK1), p38, initiation factor (IF)2α, and activating transcription factor (ATF)4 (reviewed in [94,95,96]).

### 4.4. Neurotrophic Effects

After withdrawal of cell growth supplement from the culture medium of primary rat hippocampal neurons, administration of activin A promotes cell survival. Administration of nicardipine, a Ca^2+^ channel blocker, inhibits the neurotrophic effect of activin A, as does treatment with cycloheximide, a protein synthesis inhibitor. This indicates that increased expression of voltage-gated Ca^2+^ channels is one of the neuronal responses induced by activin A [97]. Intracerebroventricular (ICV) infusion of activin A mitigates the loss of dopaminergic neurons in the substantia nigra of PD model mice induced by subcutaneous injections of 1-methyl-4-phenyl-1,2,3,6-tetrahydropyridine (MPTP). These neuroprotective effects are observed after 8 days and 8 weeks [98]. Neurodegeneration induced by ICV injection of KA in mice is mitigated by a 72-h ICV infusion of activin A that is initiated 48 h after KA injection. In a control experiment with an identical timeline, ICV injection of saline, followed by ICV infusion of activin A, increases neuronal proliferation, indicating that activin A has neurotrophic effects in both pathological and physiological conditions. The same study also demonstrates that activin A increases the number of neural stem cells, neural precursor cells, and proliferating immature neuroblasts in several brain regions, including the dentate gyrus and hippocampus [99]. In a rat model of Huntington’s disease (HD) where excitotoxic lesions are formed by intrastriatal infusion of quinolinic acid for seven consecutive days, daily intrastriatal administration of activin A leads to significantly attenuated degeneration of striatal interneurons and projection neurons after just one week [100]. In a different mouse model of HD, namely YAC128 transgenic mice, overexpression of activin A protein restores striatal neuronal excitatory N-methyl-D-aspartate receptor (eNMDAR) signaling to levels observed in control mice, which attenuates decline in motor learning and normalizes markers of reduced striatal neuronal health [101]. Adding exogenous activin A to murine brain explants exhibiting inflammasome activation leads to increased myelination of axons [102]. Similar activin A-dependent myelination is observed as a result of physical exercise in a rat model of stroke [103].

Transfection with activin A protects cultured human SH-SY5Y neuroblastoma cells against apoptosis induced by nutrient serum withdrawal from their growth medium. Additionally, activin A transfection is protective when these cells are treated with either 6-hydroxydopamine (6OHDA), a neurotoxin known to induce parkinsonism, or linsidomine (SIN-1), a peroxynitrite donor [104]. While this in vitro study is indicative of the neuroprotective effects of activin A on human neurons, the neurotrophic effects of activin A in the human brain or on human induced pluripotent stem cell (iPSC)-derived neurons have not yet been elucidated, and further studies are required.

## 5. CSF-1

### 5.1. Overview of Structure and Function

Colony-stimulating factor (CSF)-1, alternatively referred to as macrophage colony-stimulating factor (MCSF), is an 85 kDa homodimeric protein composed of two 43 kDa monomers (reviewed in [105,106]). It belongs to the hematopoietic cytokine family [107]. Murine CSF-1 (mCSF-1) and human CSF-1 (hCSF-1) display 60% overall transcript homology and 80% amino acid sequence homology within the biologically active N-terminal region (reviewed in [108,109]). This structural similarity allows recombinant hCSF-1 to elicit a response in murine cells and tissues [110,111]. In the periphery, CSF-1 is mainly produced by mesenchymal stem cells in the bone marrow, myometrial cells in the uterus, endothelial cells, fibroblasts, and mononuclear phagocytes [112,113,114]. It acts as a growth factor for macrophages and regulates osteoblast and osteocyte functions (reviewed in [115,116]). CSF-1 mRNA is also expressed in the placenta, and this cytokine is thought to regulate placental growth and differentiation [117]. Within the CNS, CSF-1 has been established as a promoter of microglial survival and proliferation (reviewed in [118,119]). Particularly, in murine models of stroke and multiple sclerosis, CSF-1 has been shown to drive microglial activity that may exacerbate these pathologies [120,121]. Although the actions of CSF-1 on neurons remain to be fully characterized, it exhibits certain neurotrophic and protective activity toward this cell type (reviewed in [122]).

### 5.2. Expression and Secretion by Microglia

In the CNS, microglia are both the primary targets and the main source of CSF-1 (Table 1) [28,122,123]. In response to the injection of glycated albumin, a protein associated with diabetic retinopathy, cultured rat retinal microglia upregulate CSF-1 secretion and increase CSF-1 receptor (CSF1R) expression [124]. The authors hypothesize that the binding of CSF-1 to overexpressed CSF1R on microglia leads to autocrine signaling and further production of CSF-1. Cultured primary human microglia have been shown to constitutively secrete CSF-1, albeit at lower levels compared to cultures of primary human astrocytes [123].

Expression of CSF-1 protein by rat microglia has been confirmed in vivo following brain damage induced by stereotaxic injection of ethanol into the striatum. CSF-1 production is particularly elevated during the later phases of this brain injury model when necrosis of striatal neurons is observed [125]. Overall, in vitro and in vivo evidence indicates that murine microglia can express CSF-1 mRNA and secrete this protein. In vitro studies with human microglia demonstrate expression and secretion of CSF-1; however, in vivo expression of this protein by human microglia has yet to be confirmed.

### 5.3. Neuronal Targets

CSF-1 exerts its effects by binding to the transmembrane CSF1R, classified as a class III intrinsic tyrosine kinase growth factor receptor (reviewed in [126]). In the resting state, CSF1R is in an inactive autoinhibitory conformation. Upon CSF-1 binding to its extracellular immunoglobulin-like domain, CSF1R shifts to an active state. Activation of CSF1R involves exposure of the autoinhibitory juxtamembrane region to the cytoplasm, resulting in the receptor adopting an active extended conformation [127]. In the periphery, CSF1R is primarily expressed in osteoclasts, macrophages, and several different types of placental cells (reviewed in [122,128]). Fetal Hofbauer cells, the resident macrophages of the placenta, also express CSF1R [129]. In the human and murine CNS, CSF1R is prominently expressed by microglia [130,131,132]. Additionally, there is evidence suggesting the expression of this receptor by neurons under physiological and pathological conditions [133,134].

In situ hybridization demonstrates CSF1R mRNA expression by cultured mouse primary hippocampal neurons [134]. In comparison to tissues harvested from uninjured brains, neurons in primary mixed glia-neuron cultures from mice brains with cerebral focal ischemic lesions express greater levels of CSF1R mRNA, indicating upregulation of this receptor in response to injury. Furthermore, CSF1R antibodies immunostain neurons in both healthy and injured murine cerebral cortex, confirming the in vivo neuronal expression of this receptor [135]. Following subcutaneous KA injection that elicits injury to murine brains, CSF1R mRNA expression is prominently upregulated in the neuron-dense pyramidal cell layer [134]. CSF1R expression is also upregulated in murine cortical neurons in response to surgically induced ischemic lesions [136]. In comparison to the demonstrated expression of CSF1R by murine neurons isolated from healthy cortices [42], a relatively low CSF1R FPKM value of 0.5 has been reported for human cortical neurons (Table 1). This may indicate low baseline expression of CSF1R under physiological conditions, but further research confirming these findings is required [28]. The binding of CSF-1 to CSF1R expressed by primary murine hippocampal neurons upregulates phosphorylation of neuronal cAMP response element-binding protein (CREB) [134]. The CREB signaling pathway has already been linked to improved neuronal survival in mice and, therefore, could be responsible for the neurotrophic effect of CSF-1, but this hypothesis will require experimental proof in both mice and humans [134,137].

### 5.4. Neurotrophic Effects

Neurotrophic effects of CSF-1 have been demonstrated both in vitro and in vivo. Incubating cultured rat cerebellar Purkinje neurons with hCSF-1 improves their survival. After seven days, up to 80% of cells exposed to hCSF-1 survive, whereas the survival rate of control neurons is as low as 30% [138]. Michaelson et al. [116] show that cultures of primary rat neurons treated with hCSF-1 contain more viable cells than control neuronal cultures after five days. The observed difference is greatest in hippocampal neuron cultures, where exposure to hCSF-1 leads to 6.6-fold higher neuronal cell counts in comparison to control cultures, with a less pronounced but similar protective effect seen in cortical neuron cultures. In comparison, this difference is only 3.4-fold in cultures of hypothalamic neurons. Furthermore, 93% of cortical neurons cultured with hCSF-1 display outgrowth of cellular processes, whereas less than 5% of neurons in the control cultures exhibit such outgrowth. Interestingly, the maximal neurotrophic effect of CSF-1 in rat hippocampal neurons is achieved at 1 ng/mL but a ten-times higher concentration of this cytokine is needed to induce the maximal protective response in cortical neurons. In summary, exposure of cultured rat neurons isolated from different brain regions to hCSF-1 results in improved cell survival, proliferation, and neurite outgrowth, helping establish the neurotrophic role of this cytokine [116].

While there is a lack of evidence for the protective actions of hCSF-1 on human neurons, the neurotrophic activity of this cytokine has been studied in murine tissues. For instance, in vivo deletion of CSF1R in murine forebrain neurons leads to their increased susceptibility to excitotoxic injury caused by KA injections. In comparison to control littermates, CSF1R-knockout mice show reduced phosphorylation of CREB in response to hippocampal neuron death induced by injection of KA into the left hippocampus [134]. Moreover, neuronal death in this CSF1R-knockout mouse model of hippocampal excitotoxic injury is more widespread, affecting more areas of the hippocampus in comparison to WT animals injected with KA. These same hippocampal injuries in CSF1R-knockout mice are also twice as likely to be fatal compared to control animals, and the surviving mutant mice display more extensive neurodegeneration indicating the crucial role of CSF-1 in the compensatory response to excitotoxic brain injury [134]. However, the neuroprotective function of CSF-1 is not limited to KA-induced injuries. Delivery of exogenous microencapsulated hCSF-1 to the site of injury facilitates neuronal survival and reduces infarct size resulting from surgically induced ischemic lesions in the murine cerebral cortex. Intraperitoneal injection of hCSF-1 in mice two weeks prior to the ischemic injury also provides similar protection. This suggests the involvement of the CSF-1/CSF1R signaling pathway in the rescue of injured neurons [136].

The critical role of CSF-1 in neural development has been demonstrated by using osteopetrotic (op/op) mice. These null mutant animals do not produce functional CSF-1 in any cell type. In comparison to control animals, op/op mice display sensory deficits such as failure to respond to simple external stimuli, which is attributed to the lack of CSF-1. They also present electrophysiological abnormalities, such as deficits in afferent information processing in the visual cortex, measured by intracortical recordings of visual evoked potentials. Subcutaneous injection of hCSF-1 in op/op mice largely prevents the development of these electrophysiological abnormalities, providing strong evidence for a direct role of CSF-1 in neural development [116]. Even though hCSF-1 is used, all the currently available studies are performed in murine models; therefore, further research into the responses of human cells and tissues to hCSF-1 is required.

## 6. Interleukin-34

### 6.1. Overview of Structure and Function

Interleukin (IL)-34 is a homodimeric glycoprotein consisting of two 39 kDa monomers that is classified as a hematopoietic cytokine (reviewed in [139,140]). First identified in 2008, IL-34 acts complimentary to CSF-1 as an alternative ligand for CSF1R (reviewed in [141]). Due to its relatively recent discovery, many aspects of IL-34 biological activity remain poorly understood; however, its neurotrophic and protective effects in the CNS have been observed. Murine IL-34 (mIL-34) and human IL-34 (hIL-34) display 71% amino acid sequence homology [140]. IL-34 exhibits some cross-species reactivity, albeit limited. Human and murine IL-34 do not cross-react, but they can both activate porcine CSF1R [140,142,143]. Despite sharing only 26% DNA sequence homology and 11% amino acid sequence identity with CSF-1, IL-34 has a very similar overall three-dimensional structure and binds the same receptor as CSF-1 (reviewed in [140,144,145]).

In the periphery, IL-34 mRNA is most highly expressed in the keratinocytes of the skin, where it contributes to the development and maintenance of Langerhans cells, a subtype of dendritic cells, that perform immune functions in the epidermis [146,147]. It also promotes osteoclast differentiation from peripheral blood mononucleated cells and bone marrow-derived cells, indicating a role in bone resorption [148]. IL-34 protein has been detected in other organs, including lung, kidney, intestine, and spleen (reviewed in [140,147,149,150,151]). Additionally, tumor-associated macrophages both express and functionally respond to IL-34 in human colon cancer [152]. In the CNS, IL-34 mRNA is expressed at high levels in the brain, particularly in the cerebral cortex, hippocampus, and striatum [142,146,147,153]. Neurons are the primary CNS source of IL-34, which is crucial for the development and maintenance of microglia [146,153,154]. In the context of neuropathologies such as AD, IL-34 facilitates microglial clearance of soluble oligomeric Aβ by inducing insulin-degrading enzymes [155]. In the aging mouse brain, IL-34, but not CSF-1, supports an autophagy-dependent protective microglia population [156]. Additionally, it participates in the maintenance of the blood brain barrier (BBB) by restoring the expression of tight junction proteins by brain endothelial cells [157]. Direct evidence of neuroprotective and neuron survival-promoting properties of IL-34 has also been collected [134,158].

### 6.2. Expression and Secretion by Microglia

Murine surveying microglia appears to express IL-34 mRNA at low levels, while expression by human microglia appears to be at very low or undetectable levels (Table 1). Notably, the analysis of frontal lobe and basal ganglia tissue sections of rhesus macaques finds that microglia produce IL-34 protein under physiological conditions [159]. Furthermore, unstimulated murine macrophages originating from CD115^+^ bone marrow-derived monocytes also produce detectable IL-34 protein [160]; however, expression of IL-34 by reactive microglia and regulation of their production of this cytokine have not been studied yet.

### 6.3. Neuronal Targets

CSF1R is the primary receptor for IL-34 and is discussed in Section 5.3. In addition to CSF1R, IL-34 binds to syndecan-1 and the receptor-type protein-tyrosine phosphatase (PTP)-ζ [161,162]. Low to moderate expression of syndecan-1 inhibits CSF1R activation by IL-34, whereas overexpression of syndecan-1 stimulates CSF1R activation by IL-34 [162]. IL-34 binding to PTP-ζ results in tyrosine phosphorylation of downstream signaling molecules including focal adhesion kinase (FAK), paxillin, and G protein-coupled receptor kinase interactor 1 (GIT1)/cool-associated tyrosine-phosphorylated 1 (Cat-1) [161]. In the adult rat brain, syndecan-1 mRNA is expressed almost exclusively in the cerebellum, which may explain the low or undetectable FPKM values recorded by analyzing cortical samples [163] (Table 1). Preliminary results from Hinsinger et al. [164] show that syndecan-1 protein levels are upregulated in the cerebrospinal fluid of MS patients. However, PTP-ζ notably has the highest expression level among the CSF-1-binding proteins. In C57BL/6J mice, PTP-ζ mRNA is detected in multiple regions of the brain, including the olfactory bulb, cerebral cortex, hippocampus, thalamus, superior colliculus, cerebellum, and medulla. In the cerebral cortex, PTP-ζ mRNA is primarily expressed in the neurons of layers II, III, and V [165]. PTP-ζ mRNA has also been detected in human subcortical white matter neural progenitors [166].

In humans, IL-34 has a higher binding affinity for CSF1R than CSF-1 [140] but in mice, the opposite is true [142]. Similar to CSF-1, IL-34 binding of CSF1R expressed by primary murine hippocampal neurons upregulates phosphorylation and activation of CREB [134]. However, it is suggested that IL-34 binds CSF1R at a different site than CSF-1 due to their sequence differences. IL-34 may form hydrogen bonds when interfacing with CSF1R, whereas CSF-1 interacts with CSF1R by establishing salt bridges. This may result in IL-34 generating unique signals that are distinct from those induced by CSF-1 upon binding to neuronal CSF1R [167].

### 6.4. Neurotrophic Effects

Primary murine hippocampal neuron cultures treated with IL-34 exhibit increased phosphorylation and activation of CREB and display reduced NMDA-induced excitotoxic cell death in comparison to control cultures [134]. IL-34 also facilitates clonal differentiation of dorsal forebrain progenitor cells by inducing bipotent neuronal clones in three-dimensional murine cortical neurosphere cultures. Co-treatment with equal concentrations of IL-34 and hCSF-1 further increases this effect in a partially additive manner [158]. Co-localization of IL-34 and PTP-ζ has been demonstrated on mature murine cortical neurons, indicating a potential role of this interaction in the mediation of higher cognitive functions [161]. In murine models, PTP-ζ has been shown to modulate several other neuronal functions including proliferation and migration [168]; however, it is unknown whether binding of IL-34 to this receptor triggers these cellular responses.

Recombinant IL-34 administered by intraperitoneal injection to FVB/N mice significantly reduces neuronal cell death in the pyramidal layer of the hippocampus following subcutaneous injection of KA. IL-34 protects against neurotoxicity, even when delivered 2 or 6 h after KA-induced injury. The neuroprotection offered by IL-34 is comparable to that of systemically administered recombinant hCSF-1 in the same murine model of neuronal damage [134]. Moreover, IL-34 promotes the survival and differentiation of neural progenitor cells in CSF-1-deficient op/op mice. These animals have significantly fewer apoptotic cortical neuronal precursor cells than CSF1R-knockout mice expressing IL-34, indicating that this cytokine has a pro-survival effect by activating CSF1R signaling [158]. Knowledge of the CNS functions of IL-34 in humans is limited, which necessitates further research. The reported elevated levels of syndecan-1 in the cerebrospinal fluid of MS patients may potentially indicate either a protective role where syndecan-1 expression is upregulated in response to pathological conditions or its neurotoxic role in which syndecan-1 signaling contributes to disease progression [164]. Given the neurotrophic effects of IL-34 that have been observed in vitro and the known downstream signaling of CSF1R, syndecan-1, and PTP-ζ in murine models, the in vivo activities of this cytokine should be explored further to identify potential new treatments for neuropathologies associated with neuronal injury.

## 7. Growth/Differentiation Factor-15

### 7.1. Overview of Structure and Function

Growth/differentiation factor (GDF)-15, also known as macrophage inhibitory cytokine (MIC)-1, is a 30 kDa disulfide-linked homodimer belonging to the TGF-β superfamily of proteins [169]. The 112-amino acid mature GDF-15 is cleaved from a 308-amino acid precursor chain, which also contains a 29-amino-acid signal peptide and 167-amino-acid pro-domain (reviewed in [170]). GDF-15 was discovered by Bootcov et al. [169] and it has been mostly studied in the context of peripheral inflammation and diabetes. In the CNS, its modulation of appetite and food intake has made GDF-15 a molecule of interest in weight loss research [171,172,173], and its neurotrophic effects remain relatively unexplored in comparison. Human GDF-15 (hGDF-15) shares only 70% amino acid sequence homology with mouse GDF-15 (mGDF-15) and rat GDF-15 (rGDF-15), the lowest of any member in the TGF-β superfamily. The amino acid sequences of mGDF-15 and rGDF-15 are highly similar, but not identical [174]. Unlike hGDF-15, which is regulated by a strong promoter, mGDF-15 is governed by a poised promoter characterized by simultaneous histone modifications associated with both activation and repression (reviewed in [175,176]). Humans instead have a poised enhancer near the promoter. These dissimilarities indicate differences in transcriptional regulation and suggest that hGDF-15 expression is controlled by elements not active in mice [176]. Despite these differences, hGDF-15 exhibits cross-reactivity in mice. Obese WT mice see reductions in plasma leptin and insulin after subcutaneous injections of hGDF-15 daily for 28 days [173]. GDF-15 protein is constitutively expressed at low levels in the periphery, with serum concentrations ranging from 0.2 to 1.2 ng/mL [177]. GDF-15 mRNA is expressed in numerous tissues including the kidney, pancreas, prostate, colon, bladder, stomach, liver, and gallbladder, with the highest expression observed in the placenta (reviewed in [178,179,180,181,182]). Expression of GDF-15 protein in humans increases with age but can also be upregulated in response to pathological conditions such as inflammation and cancer [183,184,185]. In the peripheral nervous system, GDF-15 is thought to be a neurotrophic factor since both GDF-15 mRNA and protein are upregulated following dorsal root ganglion (DRG) nerve lesions in mice [186]. Furthermore, exogenous GDF-15 accelerates sensory recovery in rats following sciatic injury [187]. Expression of this cytokine in the CNS is thought to be associated with various pathological conditions [188]. For example, GDF-15 protein secretion is increased after cerebral stroke [189,190] and processing of GDF-15 into the mature form occurs at an increased rate in AD patients [191]. Animal studies demonstrate that GDF-15 regulates food intake, energy expenditure, and body weight by acting on GDNF family receptor α-like (GFRAL) in the brainstem [192]. Specifically, the binding of GDF-15 to hindbrain GFRAL has anti-obesity actions in mice by reducing food consumption [173]. The effects of GDF-15 on food intake and obesity are being extensively researched, but its potential neurotrophic effects remain largely unexplored.

### 7.2. Expression and Secretion by Microglia

Transcriptomic analysis shows high expression of GDF-15 in murine microglia, while there may be only low levels of this cytokine present in human cells (Table 1). In THP-1 human monocytic cells, which are often used as microglia models, treatment with saturated fatty acids upregulates the expression of GDF-15 mRNA and secretion of GDF-15 protein [193]. Microglia in adult C57BL/6 mouse brains secrete GDF-15 in response to surgically induced transient MCAO [194]. A study using male Wistar rats shows that microglia upregulate the expression of GDF-15 mRNA following cryogenic lesions in the parietal cortex [195]. Further research is required to discover the factors controlling in vivo expression and secretion of GDF-15 by human microglia.

### 7.3. Neuronal Targets

GDF-15 signaling occurs through GFRAL in conjunction with rearranged during transfection (RET) transmembrane tyrosine kinase coreceptor. GDF-15 binds GFRAL prior to the recruitment of RET, which interacts with a high-affinity conformational epitope on the C1-C2 cysteine-rich domain of GFRAL [173]. The proposed model for GDF-15-induced signaling describes one GDF-15 homodimer binding two GFRALs and two RETs, forming a heterohexameric complex. This leads to the activation of the RET protein kinase and subsequent engagement of downstream signaling pathways such as protein kinase B (Akt) and extracellular signal-related kinases (ERK) [188,196]. GFRAL was initially thought to be exclusively expressed in the CNS; however, recent research has detected GFRAL protein in the peripheral tissues of mice, including the liver, small intestine, kidney, and muscle [188]. Low or undetectable levels of GFRAL mRNA expression by most murine and human CNS cell types in the cortex, including neurons (Table 1), have been reported [28,42]. It is well documented that GFRAL protein is present in the hindbrain of mice, namely the area postrema and nucleus of the solitary tract [173]. GFRAL mRNA is expressed in human brains in the area postrema and nucleus of the solitary tract, and recent research has discovered its expression in the murine arcuate nucleus and pyramidal cell layers of the medial prefrontal cortex and hippocampus [171,188]. This indicates a need for future research to determine if human neurons in these locations express GFRAL. The binding of GDF-15 to GFRAL induces phosphorylation and activation of RET, Akt, ERK, phospholipase C (PLC)-γ1, and induction of Fos [171,173,192,197]. Dissimilar to other members of the TGF-β family, there is no evidence for GDF-15 modulating SMADs [173]. Activation of Akt may be a key factor in the prevention of neuronal apoptosis in neurotoxic environments [198].

### 7.4. Neurotrophic Effects

Primary rat cerebellar granule neurons (CGN) cultured in a low potassium environment, known to induce apoptosis, are protected by pre-treatment with recombinant hGDF-15. In this experiment, GDF-15 activates Akt and suppresses ERK leading to reduced c-Jun phosphorylation, inhibition of ROS generation, and promotion of neuronal survival [198]. In another study employing primary midbrain cultures from rats, GDF-15 promotes the survival of dopaminergic neurons both in the absence of any toxic factors and under conditions of iron intoxication, indicating that this growth factor supports the survival of both normal and damaged rat neurons in cell cultures [199]. In an in vitro mouse model of PD GDF-15 exerts a neuroprotective effect, rescuing primary cultures of ventral midbrain neurons from GDF-15 knockout mice following treatment with 6OHDA [200]. In vivo research using adult rats finds that GDF-15 administered into the lateral ventricle prevents the death of nigral dopaminergic neurons induced by 6OHDA injection into the left medial forebrain bundle (MFB) [199]. In GDF-15 knockout mice, the lack of this protein exacerbates dopaminergic neuron death after injection of 6OHDA into the right MFB in comparison to WT mice injected with this neurotoxin [200]. Another study using GDF-15 knockout mice finds that these animals display progressive postnatal losses of motoneurons highlighting the neurotrophic effect of this protein during neurodevelopment [201]. Transfection of SH-SY5Y human neuronal cells with plasmid cloning DNA (pcDNA)-GDF-15 elevates GDF-15 mRNA and protein compared to control pcDNA transfected as well as untreated samples. Such an overexpression of GDF-15 reverses the upregulation of TNF, IL-6, IL-1β, and IL-8 caused by exposure of SH-SY5Y cells to the AD-related Aβ_25-35_ aggregates. GDF-15-overexpressing neuronal cells display a reduction in oxidative stress markers, including ROS, malondialdehyde, superoxide dismutase, and glutathione, resulting in protection against Aβ_25–35_-induced apoptosis [202]. In a different study, GDF-15 is shown to suppress Aβ_42_-induced apoptosis in SH-SY5Y neuronal cells by activating the Akt/GSK-3β/β-catenin signaling pathway [203]. When SH-SY5Y neuronal cell death is induced by rotenone, a toxin that triggers biochemical changes similar to those seen in PD, transfection of cells with a GDF-15 overexpression plasmid mitigates the toxic effects by decreasing ROS generation, mitochondrial damage, and apoptosis [204]. Overall, there is sufficient evidence identifying GDF-15 as a cytokine that should be studied further as a neurotrophic factor with potentially beneficial effects in human neurodegenerative diseases. Due to the recent discovery of GFRAL in the medial prefrontal cortex, hippocampus, and arcuate nucleus of mice, new studies should also aim to ascertain the effects of GDF-15 in these human brain regions.

## 8. Fibroblast Growth Factor 2

### 8.1. Overview of Structure and Function

Fibroblast growth factor 2 (FGF-2), a member of the FGF protein family, is a heparin-binding polypeptide [205]. It exerts pleiotropic effects on tissues such as the brain, blood vessels, lungs, muscles, bones, eyes, and skin (reviewed in [206]). Murine and human FGF-2 proteins are 95% homologous (reviewed in [207,208]). FGF-2 is one of several ligands for fibroblast growth factor receptors (FGFRs) 1–4 (reviewed in [209]). All four human FGFRs (1–4) exhibit high genetic similarity to their murine counterparts, with gene percent identities of 85%, 90%, 85%, and 86%, respectively [210]. Due to these similarities, cross-species reactivity is possible and has been previously observed. For example, in the AD APP/PS1 mouse model, injecting AAV2/1 viral vectors carrying human FGF-2 cDNA into the hippocampus reduces amyloid β (Aβ) deposition [211]. Furthermore, in rats with hindlimb paralysis from spinal cord transection, injecting murine FGF-2 both rostral and caudal to the epicenter of the lesion immediately after injury improves locomotor function when compared to animals receiving vehicle solution only, which remain paralyzed throughout the six-week experimental period [212]. Due to such interchangeable application of FGF-2 from different species, studies frequently do not mention the species origin of the protein used. In the periphery, FGF-2 induces angiogenesis, promotes smooth muscle cell growth, and maintains embryonic stem cells. It also supports the survival, migration, and neurite outgrowth of sensory neurons in mouse models (reviewed in [213,214,215,216]). FGF-2 has multiple functions in the development and maturation of the CNS (reviewed in [217,218]). For example, in murine neonates, FGF-2 stimulates DNA synthesis in the dentate hilus of the hippocampal region and the forebrain subventricular zone (SVZ). The increased DNA synthesis reflects the increased proliferation of neural precursor cells [219]. Additionally, FGF-2 promotes neurogenesis by regulating the proliferation and differentiation of multipotent neural progenitors within neurogenic niches of the adult murine brain. In particular, it has been shown to act in the SVZ and the subgranular zone of the hippocampal dentate gyrus (reviewed in [220,221,222]).

### 8.2. Expression and Secretion by Microglia

In adult mice, the highest mRNA expression for FGF-2 is in the mammary gland, ovary, and lungs [223]. Activated cultured macrophages of the same species stimulated with interferon (IFN)-γ also express FGF-2, as detected by reverse transcription polymerase chain reaction (PCR) [216]. Moreover, primary murine Schwann cells release FGF-2 protein into their conditioned media, which is confirmed by enzyme-linked immunosorbent assay (ELISA) [224]. In humans, the primary sources of FGF-2 protein, according to proteomic analysis, are glandular epithelial cells in eccrine sweat glands, the ascending loop of Henle, and skin adipocytes [225]. Transcriptomic analysis reveals that murine and human microglia typically express low or undetectable levels of FGF-2 under physiological conditions (Table 1). However, FGF-2 protein is present in the supernatant of both stimulated and unstimulated primary murine microglia, as determined by ELISA [226].

The co-localization of microglia markers and FGF-2 within the CNS has been shown in the murine experimental autoimmune encephalomyelitis (EAE) model of MS by employing antibodies against FGF-2 and microglia-specific fluorescein isothiocyanate-labeled *Griffonia simplicifolia* isolectin B4 (GS-IB_4_), as well as antibodies against microglial complement receptor 3 (CR3) [227]. Immunohistochemical staining also shows elevated FGF-2 protein in the postmortem cerebral cortex tissues of MS patients compared to healthy controls. Double immunostaining indicates that FGF-2 is primarily associated with reactive microglia and macrophages in MS lesions. The intensity of FGF-2 staining in these cells varies by lesion stage, with lower levels in inactive areas and higher in regions with active remyelination [228]. Furthermore, exposure of N9 murine microglial cells to hypoxic conditions increases FGF-2 protein levels in both conditioned medium and cellular lysates compared to cells maintained under normoxic conditions [229].

### 8.3. Neuronal Targets

FGFRs are transmembrane proteins that feature an extracellular portion with three immunoglobulin-like domains, a single helix in the transmembrane region, and an intracellular tyrosine kinase domain (reviewed in [230,231]). All four FGFRs activate similar downstream signaling pathways but several subtypes of this receptor exist including 1b, 1c, 2b, 2c, 3b, 3c, and 4, which all display differential preferences towards specific ligands [232].

In mice, FGFR1 and FGFR2 mRNAs are highly expressed in most tissues, while FGFR3 and FGFR4 mRNAs are more selectively expressed [233]. In humans, immunohistochemical staining shows variable expression levels of FGFR 1–4 proteins in adult tissues, with FGFR1, FGFR3, and FGFR4 expressed in a wide variety of tissues; however, FGFR2 is abundantly expressed only in the stomach and prostate [234]. Transcriptomic analyses reveal the mRNA expression of all four FGFRs in murine and human neurons, microglia, and astrocytes at varying levels [28,42]. Cultured primary murine cortical neurons express mRNAs of all four FGFRs at low-to-intermediate levels. Human cortical neurons have relatively high mRNA expression for FGFR2 only with low-to-undetectable levels for the other three FGFRs (Table 1). In situ hybridization also reveals that cortical projection neurons in adult mice express FGFR1 and FGFR2 mRNA [235]. Additionally, PCR demonstrates the presence of FGFRs 1–3 mRNAs in neurons across several regions of the rat brain, including the forebrain of postnatal day seven rats and the embryonic hippocampus and midbrain. Furthermore, mRNAs for FGFRs 1–4 are present in CGN from day seven postnatal rats [236]. In human postmortem brain samples, in situ hybridization and immunohistochemistry illustrate FGFR1 presence in hippocampal pyramidal neurons while the presence of FGFR2, FGFR3, and FGFR4 proteins are detected in the human anterior cingulate gyrus neurons [237,238]. Notably, postmortem brains from patients with Lewy body disease—a neurodegenerative disorder characterized by abnormal protein aggregates in the CNS—show an increase in FGFR3-positive neurons compared to healthy control brains, which is revealed by immunohistochemistry [238].

Once activated, FGFRs engage several intracellular signaling pathways, such as mitogen-activated protein kinase (MAPK), phosphatidylinositol 3-kinase (PI3K)-Akt, PLC*γ*, STAT, and nitric oxide-cyclic guanosine monophosphate-protein kinase G (NO-cGMP-PKG) (reviewed in [232,239,240]). FGF-2 exerts neurotrophic and neuroprotective effects on cultured murine CGNs by reducing spontaneous as well as alcohol-induced death of these cells. Inhibitors of the NO-cGMP-PKG signaling eliminate these effects, indicating that this pathway is at least partly responsible for the neuron survival-promoting activity of FGF-2 [239]. Additionally, FGF-2 in an FGFR1-dependent manner supports cell survival and promotes neurite outgrowth in cultured CGNs along with inducing phosphorylation of either p44 or p42 MAPKs, or both. This indicates that MAPKs may mediate the neuroprotective and growth-promoting effects of FGF-2 [240]. In addition to FGFRs, FGF-2 also binds to heparan sulfate proteoglycans (HSPGs) that reside on the cell surface and within the extracellular matrix (reviewed in [241,242]). HSPGs serve as co-receptors that facilitate the binding of FGF-2 to FGFRs 1–4 [243].

### 8.4. Neurotrophic Effects

Culturing neurons in serum-free, low insulin medium minimizes confounding factors when assessing the neurotrophic effects of proteins. FGF-2 under such cell culture conditions significantly improves the survival of hippocampal neurons from embryonic day 18 rats [244]. In a different study, investigating the effects of FGF-2 on neural survival, Sprague-Dawley rats receive a 14-day FGF-2 infusion into the right lateral ventricle through a cannula; two days after cannula implantation, the fimbria-fornix is transected. FGF-2 treatment rescues 66% of cholinergic neurons in the medial septum and 74% in the diagonal band of young adult rats, with protection rates in older adult rats at 45% and 47%, respectively. In a separate experiment from the same study, young adult rats undergo a fimbria-fornix transection, followed by the implantation of a cannula two days later. This cannula delivers FGF-2 for 12 days, rescuing 74% of neurons in the diagonal band while having no effect on medial septal neurons. This demonstrates the neuroprotective effects of FGF-2 on neurons damaged by axonal transection [245]. In a different model, albino rats receiving intravitreal or subretinal FGF-2 injections two days prior to constant light exposure show reduced retinal damage assessed by the thickness of the outer nuclear layer as a marker for photoreceptor loss [246]. Another study finds that rats receiving intranasal FGF-2 one hour before TBI display improved neuron survival at the injury site and reduced neurological deficits as indicated by modified neurological severity scores [247]. Finally, in the AD APP/PS1 mouse model, hippocampal injections of AAV2/1 viral vectors carrying FGF-2 cDNA enhance spatial learning, as measured by the radial arm water maze test, and increased neurogenesis in the subgranular zone [211].

FGF-2 stimulates the growth and proliferation of cultured human SH-SY5Y neuron-like cells [248]. Rotenone induces apoptosis in these cells; however, FGF-2 pretreatment significantly mitigates this injury as demonstrated by Hoechst staining and terminal deoxynucleotidyl transferase dUTP nick end labeling (TUNEL) assay [249]. Similarly, FGF-2 treatment inhibits NO-induced apoptosis in the same cells, as confirmed by the TUNEL assay [250]. It can be concluded that the available evidence from both murine and human models indicates that FGF-2 can be released by microglia to act as a neurotrophic molecule supporting the growth, survival, and differentiation of neurons in the CNS. Therefore, using FGF-2 or facilitating its release by microglia could be considered as potential therapeutic strategy for treating neurodegenerative diseases.

## 9. Insulin-Like Growth Factor 2

### 9.1. Overview of Structure and Function

Insulin-like growth factor (IGF)-2, a 7.5 kDa member of the insulin family of proteins, regulates proliferation, growth, differentiation, and survival of various cell types in the periphery (reviewed in [251,252,253,254,255]). IGF-2 has similar effects on CNS cells and acts as a neuroprotective agent by promoting the survival of neurons [256,257,258]. Murine and human IGF-2 share 84% amino acid sequence identity [207,208]. IGF-2 can bind to and activate several receptors, including insulin receptor (INSR), insulin-like growth factor 1 receptor (IGF1R), and IGF2R (reviewed in [210,259]). Murine INSR, IGF1R, and IGF2R are 86%, 88%, and 81% homologous to their human counterparts, respectively [210]. Due to these similarities, cross-species reactivity can occur and has been previously observed. For example, in the APP/PS1 AD mouse model, infusing human recombinant IGF-2 into the lateral ventricle for seven days reduces hippocampal Aβ plaques [260].

IGF-2 mRNA is also highly expressed in murine fetal tissues [261]. In rats, IGF-2 mRNA expression drops significantly in several peripheral organs, including the liver, muscles, and lungs, during early postnatal development [262]. Correspondingly, IGF-2 protein concentration in blood decreases sharply in the first few weeks following birth [263]. The key role of this growth factor in fetal development is demonstrated by the fact that IGF-2 KO mice display significantly impaired growth during this stage compared to WT animals [264,265].

In situ hybridization localizes IGF-2 mRNA to the following 16 to 20-week-old human fetal tissues: skin, muscle, thymus, heart, lung, liver, stomach, intestine, pancreas, spleen, adrenal, kidney, costal cartilage, and eye [266]. While IGF-2 protein is expressed at high levels in human fetal tissues, expression levels vary significantly across adult human tissues [267]. According to northern blot analysis, adult skin shows the highest expression, followed by peripheral nerves and striated muscle. The kidney, colon, uterus, and stomach show undetectable to very low IGF-2 mRNA levels [262]. Carlsson-Skwirut et al. [268] confirm the presence of IGF-2 protein in postmortem adult human brain tissue free from neuropathology.

### 9.2. Expression and Secretion by Microglia

According to transcriptomic analysis, primary murine microglia from WT mice express low levels of IGF-2 mRNA, and low to undetectable levels have been reported in microglia extracted from human cortices (Table 1); however, cultured human fetal microglia have been shown to express IGF-2 mRNA and protein, confirmed by quantitative PCR and western blot experiments, respectively [269]. Notably, in human brain tissues, IGF-2 protein is not detected in microglia or macrophages. The authors suggest this discrepancy can be caused by low protein expression levels, ineffective antibody staining, or swift IGF-2 capture and degradation after it binds to IGF2R. Immunoprecipitation detects IGF-2 protein in the supernatants from IFN-γ-treated primary murine microglia and immunohistochemistry experiments reveal upregulated IGF-2 protein expression by murine microglia for 7 days following a stereotactically-induced cortical lesion [270]. Kihira et al. [271] find that the number of Iba1-positive microglia is significantly increased in human amyotrophic lateral sclerosis (ALS) spinal cords compared to controls. Confocal microscopy reveals co-localization of Iba1 and IGF-2 in rod-like microglia in the anterior horn of ALS spinal cords, a phenomenon that cannot be readily observed in the ramified microglia of controls. Notably, spinal motor neurons in ALS patients demonstrate immunoreactivity for both IGF-2 and IGF2R, while control motor neurons are positive for IGF-2, but not IGF2R.

### 9.3. Neuronal Targets

IGF1R and INSR are structurally similar, belonging to the receptor tyrosine kinase (RTK) superfamily. They form heterotetrameric complexes that contain two extracellular α-subunits and two transmembrane β-subunits, arranged as αβ-chain dimers joined by disulfide bridges (reviewed in [259,272]). INSR has two isoforms, namely INSR-A and INSR-B. IGF1R and both INSR variants can form homo- or hetero-dimers. IGF-2 exhibits varying affinities for different receptors: it has the highest affinity for IGF1R, an equal affinity for INSR-A and IGF1R/INSR-A hybrids, and the lowest affinity for INSR-B and IGF1R/INSR-B heterodimer [273]. IGF1R and INSR autophosphorylate upon ligand binding, initiating similar intracellular signaling cascades. These include the PI3K-Akt-forkhead box protein O (FOXO) pathway and the MAPK/ERK kinase-extracellular signal-regulated kinases (MEK-ERK) 1 and 2 signaling (reviewed in [259]). IGF2R belongs to the p-type lectin family and is a type-I transmembrane glycoprotein containing a large N-terminal extracellular region, a single membrane-spanning region, and a small cytoplasmic tail [274]. Unlike IGF1R and INSR, the C-terminal domain of IGF2R lacks kinase activity. Instead, IGF-2/IGF2R interaction likely leads to IGF-2 endocytosis, intracellular transport, and breakdown [259]. Chen et al. [275] demonstrate the role of IGF2R in IGF-2-mediated memory enhancement. Co-injection of IGF-2 with IGF2R-specific inhibitors eliminates memory enhancement achieved by administration of the growth factor on its own.

Both primary murine and human cortical neurons express mRNA for INSR, IGFR1, and IGFR2 (Table 1). In male Kunming mice, double immunostaining reveals the coexistence of INSR with the cholinergic neurons in the CA1 region of the hippocampus [276]. In male C57BL/6 mice, immunohistochemical staining reveals the presence of IGF1R in the ipsilateral hemisphere after either controlled cortical impact (CCI) or sham injury not involving cortical impact. In the control cortices, IGF1R is predominantly expressed by neurons, particularly in layer IV. However, following injury, the overall expression levels of IGF1R remain unchanged [277], and IGF2R immunofluorescence colocalizes with microtubule-associated protein (MAP)2, a neuronal marker, in the rat hippocampus [278]. Human studies using postmortem frontal cortex samples demonstrate the presence of INSR and IGF1R in cholinergic neurons, in both control and AD brain tissues [279]. Further examination of human brain sections reveals IGF2R immunoreactivity in neurons of the frontal cortex, hippocampus, and cerebellum. This protein is primarily observed in neuronal cell bodies and processes, including pyramidal neurons [280].

### 9.4. Neurotrophic Effects

IGF-2 significantly reduces cell death of CA1 pyramidal neurons in organotypic murine hippocampal cultures subjected to 35-min oxygen-glucose deprivation. When assessed 48 h later using propidium iodide fluorescence, IGF-2-treated neuronal cultures show a 60% reduction in cell death compared to untreated cultures [281]. Schmeisser et al. [282] demonstrate that IGF-2 restores synapse density and promotes spine maturation in cultured IκB kinase/nuclear factor-κB (IKK/NF-κB) signaling-deficient hippocampal neurons. Conditional genetic inactivation of IKK2 in forebrain neurons of adult mice leads to reduced levels of mature spines in the hippocampal CA1 region. Exogenous IGF-2 restores synapse density and promotes spine maturation in IKK/NF-κB signaling-deficient neurons within 24 h. Conversely, applying neutralizing anti-IGF-2 antibodies replicates within 24 h the synaptic defects observed in IKK2-deficient neurons in control cultures [282]. IGF-2 is also neuroprotective against corticosterone-induced damage in adult rat cortical neuronal cultures [283].

Infusion of viral vectors containing murine IGF-2 transcripts into the CA1 region of both hippocampal hemispheres promotes dendritic spine formation in aged WT mice. Furthermore, the overexpression of IGF-2 by this method in an amyloid precursor protein mouse model of AD has the same effect on spine formation and restores normal hippocampal excitatory synaptic neurotransmission [284]. Additionally, IGF-2 increases neuron numbers in a rat model of intracerebral hemorrhage (ICH) induced in male rats by injecting autologous blood into the left hippocampus. Recombinant IGF-2, when injected into the damaged area 30 min post-ICH, increases the number of neurons in the hippocampal CA1 area compared to untreated control animals. This effect is observed 14 days after the injection [285].

IGF-2 promotes neurite outgrowth in SH-SY5Y human neuroblastoma cell cultures, increasing both the percentage of neurite-bearing cells and the average neurite length. This is mediated by the activation of IGF1R, as evidenced by the use of specific IGF1R inhibitors that completely block the neurite outgrowth-promoting effects of IGF-2 [258]. Furthermore, immunohistochemistry reveals that IGF-2 treatment rescues newly formed hippocampal neurons observed in mice intraperitoneally injected with LPS [286]. Additionally, IGF-2 rescues human iPSC-derived spinal motor neurons from both SOD1G93A astrocyte-mediated toxicity and glutamate-induced degeneration when added 24 to 48 h after the toxic insult [257]. In mixed human fetal neuron-glia cultures, IGF-2 reverses neuronal death caused by coadministration of IL-1 and IFN-γ [269]. While the neurotrophic and neuroprotective effects of IGF-2 have been documented by multiple studies, the secretion of this cytokine by microglia has not been established conclusively; therefore, it is imperative to study the regulation of its expression in surveying and reactive microglia in different species before IGF-2 can be regarded as a microglia-derived neurotrophic factor.

## 10. Conclusions

Even though microglia, alongside astrocytes, are a known source of neurotrophic factors, the majority of review articles detailing this aspect of microglial contributions to CNS homeostasis highlight several proteins with already established neurotrophic activities including BDNF, GDNF, NGF, NT-3, and NT-4/5. In this review article, we chose to focus on eight different molecules that so far have not been viewed as canonical microglia-derived neurotrophic factors, but yet have sufficient evidence supporting their expression and/or secretion by microglia as well as indications of their trophic and protective effects on neurons (Figure 1, Table 2). A review of the available literature reveals that the data supporting the expression of these cytokines by murine and human microglia are highly varied (Table 1 and Table 2). For example, microglial expression of OSM and activin A has been demonstrated conclusively under diverse functional states, while the expression of several other of the considered cytokines, such as LIF and IL-34, by this cell type is understudied. Further investigation of the modulation of IL-34 expression by microglia is warranted in particular. Our review also illustrates that data demonstrating differential expression of these cytokines by microglia under various pathological conditions are beginning to accumulate. Several widely used immune stimulants, such as LPS, PMA, ATP, and IFN-γ, are shown to increase the expression of some of the considered molecules, including OSM, LIF, activin A, and IGF-2. Additionally, diverse pathologies associated with neurodegenerative diseases, infection, ischemia, and traumatic injury are linked to increased levels of OSM, LIF, GDF-15, and FGF-2 (Table 2).

In addition to microglial production of the cytokines, we also reviewed the expression of their corresponding receptors by neurons. In most cases, there is sufficient evidence of such receptor expression by at least one distinct type of murine neurons. Not surprisingly, only very limited data are available with regard to the expression of these receptors by human neurons and more generally the human CNS, which represents a knowledge gap for nearly all of the cytokines considered. We also conclude that the demonstrated neurotrophic effects for each of the eight cytokines are varied and include increased neuronal survival or proliferation, recovery of neurons after insult, enhanced neurite outgrowth, increased differentiation, and attenuation of neurodegeneration (Figure 1, Table 2). All of the considered cytokines are shown to increase neuronal survival in one or more neuronal cell models. However, after this initial similarity, the neurotrophic effects of individual cytokines begin to diversify. Recovery after insult is documented for five out of eight molecules: OSM, LIF, GDF-15, FGF-2, and IGF-2. Increased differentiation of neurons from neuronal precursors has been reported for LIF, activin A, and IL-34. Enhanced neurite outgrowth is found after the treatment of cells with OSM, CSF-1, and IGF-2. Elevated rates of proliferation, separate from increased survival of pre-existing cells, is a functional characteristic of only CSF-1 and IGF-2. Attenuation of neurodegeneration is an activity attributed to OSM, activin A, and IGF-2. Currently, it is not clear whether such partially overlapping neurotrophic activities are true characteristics of the reviewed cytokines or an illustration of the fragmented nature of our current knowledge related to these molecules as neurotrophic factors. Comprehensive studies using each of the cytokines and covering the full spectrum of neurotrophic effects are needed to answer this question.

Given the established dissimilarities between murine and human immune systems [287] and the emerging interspecies differences in microglia neuroimmune functions (reviewed in [9,288]), our review highlights this often-overlooked aspect of microglia-to-neuron signaling (Table 2). Notably, while the majority of the considered cytokines can be used interchangeably between humans and rodents, this is not the case for OSM and LIF. We further distinguish between the expression of cytokines by murine and human microglia as well as the effects of these cytokines on murine and human neuronal cells and in animal models of disease. This approach clearly identifies the expression of cytokines by human microglia and their effects on human neurons and human tissues as a significant knowledge gap that should be addressed in future experimental and clinical studies. This is particularly important if these cytokines or the receptors they interact with are considered therapeutic targets for human neurological disorders. We feel such further research is warranted due to the already available preclinical studies using in vitro and in vivo systems which demonstrate neurotrophic effects of these cytokines in models of ischemic stroke, hypoxia, nutrient deprivation, ICH, TBI, axotomy, hypokalemia, iron intoxication, immunodeficiency, steroid toxicity, inflammation, excitotoxic injury, SCI, and a variety of neurodegenerative disorders, including AD, MS, PD, and HD (Table 2). Only more comprehensive future research could establish which of these disorders could benefit from interventions using or targeting the signaling pathways triggered by each of the microglia-derived cytokines potentially possessing neurotrophic properties.

## Figures and Tables

**Table 1:** Expression of cytokines by murine and human microglia and the corresponding cytokine receptors by murine and human neurons.

Expression of Cytokines by Microglia	Expression of Cytokine Receptors by Neurons	Expression by Murine Cells (FPKM)	Expression by Human Cells (FPKM)
Oncostatin M (OSM)		209.9	3.2
	OSMRß	0.2	0.78
	LIFRß	5.1	6.5
	gp130α	8.2	18.6
Leukemia inhibitory factor (LIF)		2.3	0.1
	LIFRß	5.1	6.5
	gp130α	8.2	18.6
Inhibin ßA (INHBA)		0.1	17.7
	ACVR1A	9.5	3.5
	ACVR2A	12.0	5.3
	ACVR1B	17.5	6.0
	ACVR2B	3.6	1.6
Colony-stimulating factor (CSF)-1		198.4	9.5
	CSF1R	1.9	0.5
Interleukin (IL)-34		1.3	0.1
	CSF1R	1.9	0.5
	Syndecan-1	5.2	0.1
	PTP-ζ	45.5	20.5
Growth/differentiation factor (GDF)-15		782.9	0.1
	GFRAL	0.1	0.1
	RET	1.0	1.0
Fibroblast growth factor (FGF)-2		0.1	0.7
	FGFR1	7.4	1.0
	FGFR2	2.7	6.6
	FGFR3	9.5	0.1
	FGFR4	0.2	0.1
Insulin-like growth factor (IGF)-2		2.0	0.1
	IGF1R	6.8	1.8
	IGF2R	2.0	1.6
	INSR	8.6	3.5

**Table 2:** Microglia-derived cytokines act as neurotrophic factors by binding to established receptors.

Cytokine	Expression by Microglia	Modulators of Microglial Expression	Expression of Corresponding Receptors by Neuronal Targets	Disease Models Studied	Neurotrophic Effects Observed In Vitro (Conditions)	Neurotrophic Effects Observed In Vivo (Disease Model)
Oncostatin M (OSM)	Murine (in vitro, in vivo) Human (in vitro, in vivo)	PGE2 HIV GM-CSF LPS PMA	Murine (in vitro, in vivo*)* Human (in vitro)	SCI MS Excitotoxic injury Nutrient deprivation MCAO	**Murine:** Increased survival (excitotoxic injury) Rescue after insult (excitotoxic injury) Neurite outgrowth (nutrient deprivation)	**Murine:** Increased survival (SCI) Attenuation of neurodegeneration (MS) Rescue after insult (MCAO) Neurite outgrowth (SCI)
Leukemia inhibitory factor (LIF)	Murine (in vitro) Human (in vitro)	ATP *Borrelia burgdorferi*	Murine (in vitro, in vivo*)* Human (in vitro)	Hypoxia Oxidative stress TBI Axotomy	**Murine:** Increased survival (embryonic cells) **Human:** Neuronal differentiation (Stem cells) Neurite outgrowth (physiological conditions) Increased survival (physiological conditions) Increased survival (oxidative stress) Increased survival (hypoxia) Increased survival (TNF)	**Murine:** Rescue after insult (axotomy) Neurite outgrowth (TBI)
Activin A/INHBA	Murine (in vitro, in vivo) Human (in vitro, in vivo)	TLR2 agonists LPS and other TLR4 agonists TLR9 agonists	Murine (in vitro, in vivo*)* Human (in vitro)	Nutrient deprivation PD Excitotoxicity HD Oxidative stress	**Murine:** Increased survival (nutrient deprivation) **Human:** Rescue after insult (nutrient deprivation) Rescue after insult (PD model) Rescue after insult (oxidative stress)	**Murine:** Increased survival (PD) Attenuation of neurodegeneration (excitotoxicity) Increased proliferation (physiological conditions) Differentiation (physiological conditions) Attenuation of neurodegeneration (HD)
Colony-stimulating factor (CSF-1)	Murine (in vitro, in vivo) Human (in vitro)	Glycated albumin Ethanol	Murine (in vitro, in vivo*)* Human (in vitro)	Ischemia	**Murine:** Increased survival (physiological conditions) Increased proliferation (physiological conditions) Neurite outgrowth (physiological conditions)	**Murine:** Increased survival (ischemia)
Interleukin (IL)-34	Murine (in vitro, in vivo*)* Human (in vivo)	N/A	Murine (in vitro, in vivo*)* Human (in vitro, in vivo)	Excitotoxicity Osteopetrosis	**Murine:** Increased survival (excitotoxicity) Neuronal differentiation (physiological conditions)	Murine: Increased survival (excitotoxicity) Increased survival (osteopetrosis) Increased differentiation (osteopetrosis)
Growth/differentiation factor (GDF)-15	Murine (in vitro, in vivo*)* Human (in vitro)	Saturated fatty acids Cryogenic stimulation MCAO	Murine (in vivo) Human (in vivo)	Hypokalemia Iron intoxication PD AD	**Murine:** Increased survival (hypokalemia) Rescue after insult (iron intoxication) **Human:** Rescue after insult (AD model) Rescue after insult (PD model)	**Murine:** Increased survival (PD)
Fibroblast growth factor (FGF)-2	Murine (in vitro, in vivo) Human (in vivo)	Hypoxia	Murine (in vitro, in vivo) Human (in vivo)	Nutrient deprivation Axotomy TBI AD PD Oxidative stress Light-induced retinal damage	**Murine:** Increased survival (nutrient deprivation) Rescue after insult (Ethanol-induced stress) **Human:** Increased survival (PD model) Increased survival (oxidative stress)	**Murine:** Rescue after insult (axotomy) Increased survival (TBI) Increased survival (AD) Neuroprotection (light-induced retinal damage)
Insulin growth factor (IGF)-2	Murine (in vitro, in vivo*)* Human (in vitro)	IFN-γ	Murine (in vitro, in vivo*)* Human (in vitro, in vivo)	Oxygen-glucose deprivation Synaptic dysfunction Steroid toxicity AD ICH Excitotoxicity Inflammation	**Murine:** Increased survival (oxygen-glucose deprivation) Neurite outgrowth (synaptic dysfunction) Increased survival (steroid toxicity) **Human:** Neurite outgrowth (physiological conditions) Rescue after insult (astrocyte-mediated toxicity) Rescue after insult (excitotoxicity) Rescue (inflammation)	**Murine:** Neurite outgrowth (AD) Increased proliferation (ICH)

## Data Availability

Not applicable.

## Abbreviations

6-OHDA: 6-hydroxydopamine; Aβ, amyloid β; ACVR, activin receptor; AD, Alzheimer’s disease; Akt, protein kinase B; ALS, amyotrophic lateral sclerosis; AMPA, α-amino-3-hydroxy-5-methyl-4-isoxazole propionic acid; ATF4, activating transcription factor 4; BBB, blood brain barrier; BDNF, brain-derived neurotrophic factor; Cat-1, cool-associated tyrosine-phosphorylated 1; CGN, cerebellar granule neurons; ChAT, choline acetyltransferase; CNS, central nervous system; CREB, cAMP response element-binding protein; CSF-1, colony-stimulating factor-1; CSF1R, colony-stimulating factor 1 receptor; DAMP, damage-associated molecular pattern; DRG, dorsal root ganglion; EAE, experimental autoimmune encephalomyelitis; ELISA, enzyme-linked immunosorbent assay; eNMDAR, excitatory N-methyl-D-aspartate receptor; ERK, extracellular signal-related kinase; FGF-2, fibroblast growth factor-2; FGFR, fibroblast growth factor receptor; FPKM, fragments per kilobase of transcript per million mapped reads; GDF-15, growth/differentiation factor-15; GDNF, glial cell line-derived neurotrophic factor; GFRAL, glial cell line-derived neurotrophic factor-family receptor ɑ-like; GIT1, G protein-coupled receptor kinase interactor 1; GM-CSF, granulocyte-macrophage colony-stimulating factor; gp, glycoprotein; HD, Huntington’s disease; HIV, human immunodeficiency virus; HSPG, heparan sulfate proteoglycan; ICV, intracerebroventricular; IGF-2, insulin-like growth factor-2; IGFR, insulin-like growth factor receptor; IF2, initiation factor 2; IFN, interferon; IKK, IκB kinase; IL, interleukin; INHBA, inhibin ß_A_; INSR, insulin receptor; iPSC, induced pluripotent stem cell; JAK, Janus kinase; JNK1, c-Jun N-terminal kinase 1; K_d_, dissociation constant; KA, kainic acid; LIF, leukemia inhibitory factor; LIFR, leukemia inhibitory factor receptor; LPS, lipopolysaccharide; MAP-2, microtubule-associated protein-2; MAPK, mitogen-activated protein kinase; MCAO, middle cerebral artery occlusion; MCSF, macrophage colony-stimulating factor; MIC-1, macrophage inhibitory cytokine 1; MPTP, 1-methyl-4-phenyl-1,2,3,6-tetrahydropyridine; MS, multiple sclerosis; NGF, nerve growth factor; NMDA, N-methyl-D-aspartate; NF-κB, nuclear factor-κB; NT, neurotrophin; NO-cGMP-PKG, nitric oxide-cyclic guanosine monophosphate-protein kinase G; NOS2, nitric oxide synthase 2; 6OHDA, 6-hydroxydopamine; op/op, osteopetrotic; OSM, oncostatin M; OSMR, oncostatin M receptor; PCR, polymerase chain reaction; PD, Parkinson’s disease; PGE_2_, prostaglandin E_2_; PI3K, phosphatidylinositol 3-kinase (PI3K); PLC, phospholipase C; PMA, phorbol-12-myristate-13-acetate; PNS, peripheral nervous system; PTP-ζ, protein-tyrosine phosphatase ζ; RET, rearranged during transfection; ROS, reactive oxygen species; RTK, receptor tyrosine kinase; SCI, spinal cord injury; SIN-1, linsidomine; SOCS, suppressor of cytokine signaling; STAT, signal transducer and activator of transcription; SVZ, subventricular zone; Syn-I, synapsin-I; TBI, traumatic brain injury; TLR, toll-like receptor; TNF, tumor necrosis factor; TUNEL, terminal deoxynucleotidyl transferase dUTP nick end labeling; WT, wild-type.
